# Perception of Ultrasonic Vocalizations by Socially Housed and Isolated Mice

**DOI:** 10.1523/ENEURO.0049-19.2019

**Published:** 2019-10-14

**Authors:** Laurel A. Screven, Micheal L. Dent

**Affiliations:** Department of Psychology, University at Buffalo, the State University of New York, Buffalo, NY 14260

**Keywords:** hearing, psychoacoustics, USVs

## Abstract

It is currently unclear whether mice use their ultrasonic vocalizations (USVs) for communication purposes. It is also unknown whether mice require previous experience with USVs to understand conspecifics. There is some evidence that experience changes the perception of juvenile USVs; however, it is unclear whether similar plasticity also occurs for adult USVs. To examine whether social exposure or deprivation throughout development leads to changes in USV perception, eleven female CBA/CaJ mice were trained to discriminate between 18 USVs of three different categories using operant conditioning procedures. Mice were group housed with four females or housed individually from weaning for the duration of the experiment. Socially housed and isolated mice differed in initial training times on pure tones, suggesting isolated mice had a more difficult time learning the task. Both groups completed USV discrimination conditions quicker at the end of the testing phases relative to the beginning. The overall discrimination of USVs did not differ between the two housing conditions, but a multidimensional scaling analysis revealed that socially experienced and isolated mice perceive some USVs differently, illustrated by differences in locations of USVs on the scaling maps from the two groups. Finally, a negative correlation was found between spectrotemporal similarity and percent discrimination, and analyses support the idea that mice may show categorical perception of at least two of the three USV categories. Thus, experience with USVs changes USV perception.

## Significance Statement

The present experiment is the first to behaviorally measure changes in perception of ultrasonic vocalizations (USVs) by mice after different levels of social experience. Electrophysiological experiments showed increases in cortical spiking in maternal females in response to pup calls (Liu and Schreiner, 2007; Cohen et al., 2011), but it is unknown whether changes in neuronal activity are correlated with changes in perception. Psychophysical measurements of USV perception from awake, behaving mice with different social experiences are sorely needed. Further, USVs have historically been divided into researcher-defined categories, and the present experiment is the first to examine whether mice exhibit categorical perception of adult USVs. It is critical to ascertain the factors that influence auditory perception to further our understanding of mouse communication.

## Introduction

For vocalizations to be useful for communication, it is critical to perceive and understand the various vocal signals emitted by conspecifics. Mice emit ultrasonic vocalizations (USVs), which differ in spectrotemporal parameters (e.g., frequency, duration, and intensity). USVs are produced in same- and opposite-sex interactions by both males and females (Sewell, 1972; Scattoni et al., 2011; Hammerschmidt et al., 2012; Neunuebel et al., 2015) and are assumed to facilitate social interactions (Portfors, 2007; Okanoya and Screven, 2018).

USVs have been divided into categories based on spectrotemporal parameters (Sewell, 1972; Portfors, 2007; Scattoni et al., 2011; Hammerschmidt et al., 2012), but it is currently unknown whether these categories are meaningful to the mice. Due to the spectrotemporal variability of USVs, individual USVs could possibly have a situation-specific function in mice, where certain USVs or sequences communicate information in particular situations. To determine whether USVs have context specificity, we must first ask whether mice can discriminate between the various USVs they produce. When mice are able to discriminate between USVs, the USVs have the potential to communicate context-specific information.

Auditory processing is often examined using electrophysiological methods, such as extracellular recordings (Portfors et al., 2009). Electrophysiology does not require training, but it often yields measures that are less informative than results from awake, behaving mice (Klink et al., 2006; Heffner et al., 2008). It is impossible to determine whether differences in neural responsivity correspond to actual differences in perception by the animal. Through operant conditioning, mice are trained to be “reliable observers” (Heffner and Heffner, 2001, p 19) and researchers can make more accurate and nuanced measures of their perceptual abilities (Dent et al., 2018). For example, Neilans et al. (2014) found that mice could discriminate between different categories of USVs, with increased discrimination for spectrotemporally dissimilar vocalizations. That experiment was the first to show that mice could discriminate between USVs of different researcher-defined categories. The findings of that experiment are limited, however, because only one USV from each researcher-defined category was used. The relationship between discrimination and spectrotemporal similarity could be more complex than was reported by Neilans et al. (2014).

Perception of USVs may be affected by social experience. Neilans et al. (2014) used chronically socially isolated mice, and isolation could have led to deficits in discrimination. Mice may need to be exposed to USVs through social experience throughout development for those USVs to have a communicative function (see Chabout et al., 2015, 2016). The role of social experience with USVs on the spiking activity of neurons in the mouse auditory cortex was examined by Liu and Schreiner (2007). There were significant increases in cortical spiking activity in maternal compared to virgin female mice to pup USV playbacks. Cohen et al. (2011) extended these findings to include pup-experienced virgin females, and showed similar spiking activity as in maternal females. This increase in spiking activity arose presumably because of the behavioral relevance of these USVs to the pup-experienced virgin and maternal females compared to pup-naive virgin females. It is unclear from these findings whether there were perceptual differences to pup calls as a result of differences in spiking activity since no behavioral measurements were taken.

Social interactions could be critical for mice to discriminate vocalizations emitted by conspecifics. Social experiences of female mice are known to influence the preference for USVs and olfactory signals (Screven and Dent, 2018), but not the production of USVs (Screven and Dent, 2019). Preventing mice from learning the connections between vocalizations and context could be detrimental to their ability to perceive USVs although it does not change the production of the USVs. To investigate the effect of social deprivation on USV perception, we compared USV discrimination in socially housed and chronically isolated mice. We hypothesized that exposure to conspecifics through social housing would improve USV discrimination compared to discrimination by isolated mice. Additionally, we hypothesized that chronic social isolation would lead to irreversible perceptual deficits. There was a difference in training time between mice in the two social housing conditions in early training on pure tones, but not on later overall USV discrimination abilities, although a multidimensional analysis revealed differences in perceptual mapping of the USVs between the two groups. Finally, we were able to expand on the findings of Neilans et al. (2014) by examining the role of spectrotemporal similarity of USVs on discrimination performance, and found that the mice perceived at least some of the USVs in a categorical manner.

## Materials and Methods

### Animals

Eleven female CBA/CaJ mice were used for this experiment. These mice were divided into two groups: individually housed and socially housed. All five individually housed mice were experimentally naive. Two socially housed female mice (Js and Fr) were experimentally naive; the remaining four (Jo, Ja, Re, and Fa) were previously tested on a similar experiment investigating USV perception. That experiment was an exploratory test to examine whether mice could more easily discriminate between USVs from a familiar versus an unfamiliar mouse. The two socially housed mice that were experimentally naive provided a control for the other mice’s previous experience with the test stimuli and procedure. The individually housed mice were separated from their litter at weaning and lived alone in their home cages (30 × 19 × 13 cm) with stainless steel covers, bedding, and nesting material for the duration of the experiment. These mice did not have any social contact with any other mice, although they were not acoustically isolated from the rest of the colony. The six socially housed mice were housed in two large home cages (47 × 25.5 × 21.5 cm) with stainless steel covers, bedding, and nesting material. Socially housed mice lived in groups of four. Re, Ja, Jo, and Fa lived together in cage 1 and Fr and Js lived in cage 2, along with two mice used in other experiments.

### Apparatus

**Figure 1.**
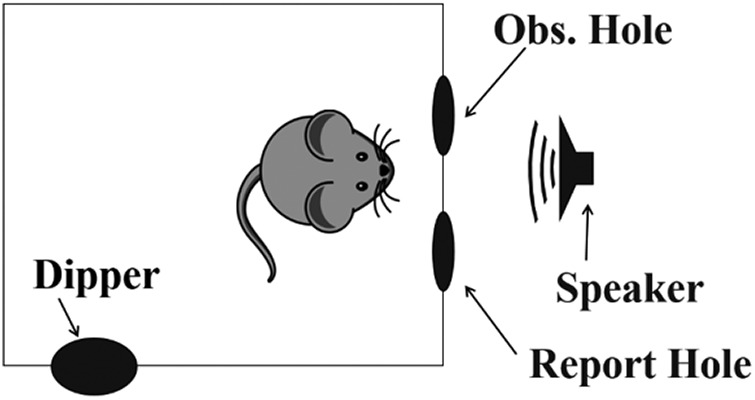
Schematic of the operant conditioning chamber. Mice were trained to nose poke into the observation (obs.) and report holes to receive reinforcement from the dipper.

The mice were tested in a wire cage (23 × 39 × 15.5 cm) placed in a sound attenuated chamber (53.3 × 54.4 × 57 cm) lined with 4-cm thick Sonex sound attenuating foam (Illbruck, Inc.). The chamber contained an overhead web camera (Logitech B910 HD) and a small 25-W white light to monitor mice during test sessions. Signals were played from an electrostatic speaker (Tucker-Davis Technologies, Model ES1). The cage also contained two nose-poke holes surrounded by infrared sensors (Med Associates Model ENV-254), and a response dipper (Med Associates Model ENV-302M-UP; Fig. 1).

### Test stimuli

Vocalizations were recorded from two female CBA/CaJ mice (Mouse K and Mouse I) who previously (approximately six months earlier) lived with the four experimentally experienced mice (two groups of four, one vocalizer per cage). Three mice lived with one vocalizer (Re, Ja, and Jo lived with Mouse K), and the remaining mouse lived with the other (Fa lived with Mouse I). The mice used in this experiment did not live with the vocalizers at any point during testing. Vocalizations were recorded using a condenser microphone [UltraSoundGate CM16/CMPA, flat frequency response (±6 dB) between 25 and 140 kHz], which was attached to the lid of the recording chamber, 8 cm above the cage. Acoustic signals traveled to an HP Pavilion 500 PC computer through an Avisoft recorder (BioAcoustics UltraSoundGate 116H, 300-kHz sampling rate with a 16-bit format). Vocalizations were analyzed with Adobe Audition CS6 on an HP Pavilion 500 PC.

The stimuli were recorded by placing one female in a home cage (18.5 × 12.5 × 29.5 cm) that contained dirty bedding from a cage mate. The cage was placed inside a recording chamber (46 × 41 × 74 cm). This chamber was lined with Sonex anechoic foam (4 cm). Vocalizations were elicited from the mice by putting a cage mate in a separate home cage 14 cm away, following a 6-h separation. Due to the high directionality of the USVs, we were able to ensure all USVs in the recording were from the desired female, as USVs from the other female were much quieter on the recording.

**Figure 2.**
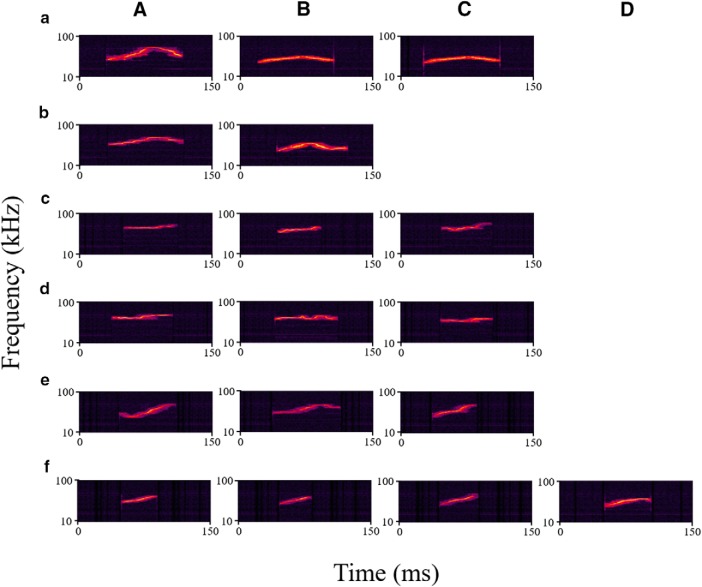
Spectrograms of all USVs used in this experiment. USVs in rows ***a***, ***c***, ***e*** were produced by Mouse K, while USVs in rows ***b***, ***d***, ***f*** were produced by Mouse I. Chevron vocalizations are depicted in rows ***a***, ***b***. Complex vocalizations are depicted in rows ***c***, ***d***. Upsweep vocalizations are depicted in rows ***e***, ***f***. ***A–D*** are different renditions of each USV.

The recordings were analyzed and we chose USVs from three categories to use as stimuli in this experiment. These categories were chevron, complex, and upsweep (Fig. 2). A total of 18 USVs were used as stimuli: five chevron, six complex, and seven upsweep USVs. Chevron USVs were characterized by an inverted U-shape in frequency modulation. They had a mean 20-kHz rise and 13-kHz fall in frequency and a mean duration of 83 ms. Complex USVs had a minimum of two changes in the direction of frequency modulation and frequency modulations of at least 5 kHz. These USVs had a mean duration of 60 ms. Upsweep USVs were characterized by an increase in frequency across the duration of the USV, with a mean total increase of 20 kHz. Upsweep USVs may show a slight decrease in frequency at the end of the USV, but the end frequency of the stimulus must be >7 kHz from the starting frequency of the stimulus. Therefore, these upsweep USVs are distinct from chevron USVs. Additionally, upsweep USVs may show a slight decrease in frequency at the beginning of the USV. The upsweep USVs had a mean duration of 51 ms. All USVs were presented at the same intensity [50-dB SPL, LAFmax (maximum intensity of a sound rising using the A-scale and fast time constant)] during the entirety of the experiment.

The stimuli were named, for example, with the nomenclature of: “I Chevron B”, where “I” refers to the vocalizer (I or K), “Chevron” refers to the category of USV (chevron, complex, or upsweep), and “B” refers to the rendition (A-D, a random assignment of different vocalizations fitting the category produced by the specific vocalizer).

### Experimental design and statistical analyses

Mice were trained using a go/no-go operant conditioning procedure on a discrimination task. The mice were tested once per day for 1 h, 7 d per week. Each mouse was tested on all 18 USVs unless they became too ill to complete the experiment. Each USV served as a background and target stimulus. Every background-target stimulus combination was tested for each mouse.

Before the mice began testing, they were trained using strict criteria for both hit rate and false alarm rate on a pure tone detection task. The training stimulus was a 500-ms 16-kHz pure tone with a 40-ms rise/fall time. Pure tones were presented two times, separated by 500 ms of silence, and mice were required to correctly respond to tones before being tested using USVs. After mice reliably detected the 16-kHz pure tone, they were transitioned to USV stimuli. To ensure that the mice understood the discrimination procedure, they were first trained to detect high-intensity target USVs while the background USV was attenuated to 0-dB SPL. When the subjects were able to detect over 80% of the targets correctly with less than a 20% false alarm rate (criterion performance), the background attenuation decreased by 5–10 dB until the same criterion performance was reached again, then the background was attenuated less, and so on. This continued until the background USV was presented at the same intensity as the target USVs, at which time the mouse was considered in the testing phase.

**Figure 3.**
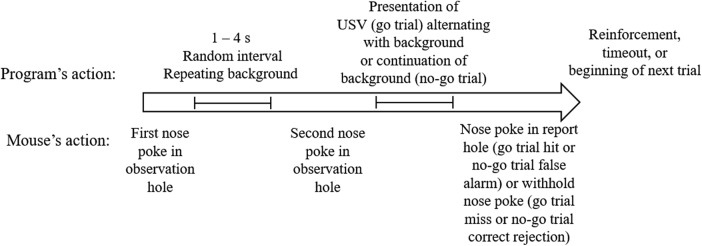
Schematic of the trial structure of the USV discrimination task.

Mice began a trial by nose poking through the left observation hole, which initiated a variable waiting interval that ranged from 1 to 4 s. During this time, one of the 18 USVs that served as the background stimulus was presented repeatedly, with a 200-ms silence interval between each presentation. Only one background USV was presented per session. After the waiting interval, one of seven possible target USVs was presented, alternating with the background two times. When the mouse was able to discriminate between the background and the target USVs, it was required to nose poke through the right report nose-poke hole within 2 s of the onset of the test stimulus. In this trial type, a “hit” was recorded when the mouse responded within the response window and the mouse received 0.01 ml of Ensure as reinforcement. A “miss” was recorded when the mouse failed to nose poke through the report nose-poke hole during the response interval. In this case, the mouse was able to move on to the next trial immediately, as no punishment (timeout) was administered when the mouse missed a target stimulus. A schematic of the trial structure is shown in Figure 3.

Experimental sessions consisted of multiple random blocks of ten trials each. Within each block of ten, seven were “go” trials and three were “no-go” trials. The sequence of go and no-go trials within each block was random except for the constraint that no more than two no-go trials were presented in a row. In the no-go trials, the repeating background continued to be presented during the response phase. These trials were required to measure false alarm rate to determine whether the mouse was guessing. When the subject nose poked in the report poke-hole during the response period, a “false alarm” was recorded and the mouse was punished with a 5-s timeout interval, during which another trial could not be initiated. When the subject continued to nose poke into the observation hole, a “correct rejection” was recorded and the mouse continued on to the next trial immediately (no timeout). In either case, no reinforcement was given. False alarm rate is a measure of how often mice randomly responded into the report hole when no target USVs were presented. Sessions were excluded from analysis when the mice’s false alarm rate exceeded 20%.

In go trials, seven of the seventeen possible target USV types were presented randomly throughout the session. When a mouse completed 20 trials for each of the first seven USV types (200 total trials including the 60 no-go trials) with a false alarm rate <20%, the mouse remained on the same background USV and the next seven USV types were used as target stimuli. Mice were not required to reach a minimum percent correct of 80% during the testing phase, as mice were not able to discriminate some targets from the background during testing. After the mouse completed 200 trials for the second group of target stimuli, they were tested on the last three USVs with the same USV serving as the background as the previous two conditions and completed 20 trials for each of the remaining 3 USVs (100 total trials, including 30 no-go trials and 10 already tested USV trials). A mouse completed a condition once all 17 target USVs had been discriminated from the background USV at least 20 times each. All mice were tested on all background-target USV combinations in a random order, and a different random order was used for each subject. Mice completed between 50 and 300 trials per session.

To determine whether there were cognitive deficits in isolated mice, we conducted a Mood’s median test to compare the number of training days required by the isolated and socially housed mice before they could begin testing. We also conducted a Mood’s median test to evaluate the relationship between condition number and number of days required to complete each condition between socially housed and isolated mice. It is important to note that condition number refers to the order the mice ran on each condition, regardless of which USV served as the background. To correct for multiple comparisons, a Bonferroni correction was applied such that significant results are denoted by *p* < 0.003. Additionally, to determine whether there were differences in discrimination of target USVs from background USVs between social and isolated mice, we conducted a Mann–Whitney *U* test on the percent correct for each target USV separately, using housing condition (social vs isolated) as the factor of comparison. A Bonferroni *post hoc* correction was applied to correct for multiple comparisons, such that significant differences are denoted by *p* < 0.003. Next, we conducted a multidimensional scaling analysis (PROXSCAL, identity), which used percent discrimination of every USV versus every other USV to determine the perceptual maps of the USVs for social and isolated female mice. To examine how each mouse contributed to the multidimensional scaling analyses, we created an individual weight graph (PROXSCAL, weighted Euclidian). To determine the relationship between discrimination performance and spectrotemporal similarity of USVs, we conducted Spearman’s rank-order correlations for social and isolated mice. Lastly, to examine whether mice differentially discriminated USVs within versus between categories, we conducted three Mann–Whitney *U* tests on discrimination performance (percent correct) for all three background USV categories (within category vs between categories) for both socially housed and isolated mice. To correct for multiple comparisons, a Bonferroni correction was applied such that significant results are denoted by *p* < 0.008.

## Results

### Number of training and testing days per condition

**Figure 4.**
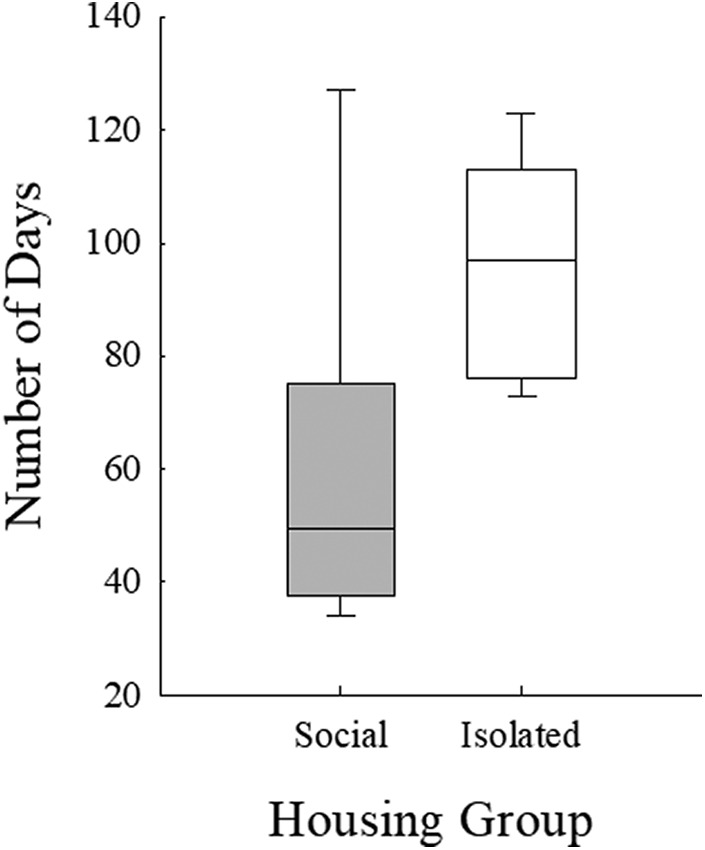
Number of days required for social (gray bars) and isolated (white bars) mice to complete pure tone detection training. Boxes represent upper and lower quartile range, horizontal lines represent median, error bars represent maximum and minimum number of days.

**Figure 5.**
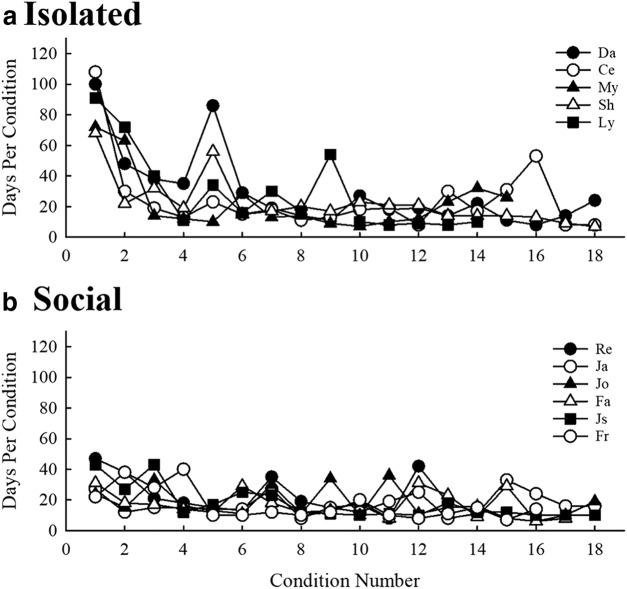
Number of days required for (***a***) isolated and (***b***) socially housed mice to complete each of the 18 background USV discrimination conditions.

A Mood’s median test revealed significant differences between socially housed mice and socially isolated mice with respect to number of days required to train on pure tone stimuli (χ^2^ = 4.412, *p* = 0.036). Socially housed mice required a median of 50 d to complete training, significantly fewer days than isolated mice, which required 97 d (Fig. 4). This effect is not the result of the four social mice (Jo, Ja, Re, and Fa) having experience with another USV perception task before the beginning of the present experiment, as the training took place before the onset of all USV discrimination tasks for all mice in both housing groups. Next, the number of days required to complete each USV discrimination condition was examined in both housing groups (Fig. 5). The results of the Mood’s median test revealed isolated mice required significantly more days to complete the first condition than socially housed mice (χ^2^ = 11.000, *p* = 0.0009; regardless of which USV served as the background). No other condition showed significant differences between mice in the two housing conditions. Thus, initially, socially housed mice performed better on the discrimination task than isolated mice, but after the first condition both groups required a similar number of days to complete each condition.

### Discrimination of target USVs

**Figure 6.**
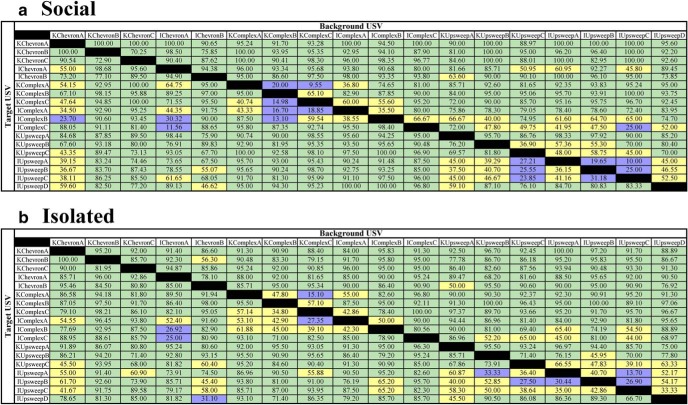
Mean percent correct discrimination values for every background USV against every target USV for social (***a***) and isolated (***b***) mice. Percent corrects ranging from 0% to 33% are shaded in purple. Percent corrects ranging from 34% to 66% are shaded in yellow. Percent corrects ranging from 67% to 100% are shaded in green.

The discrimination performance (percent correct) for all target USVs within a single background USV was compared between mice in the two housing conditions (means are shown in Fig. 6, separately for mice in each housing condition). Both groups were generally good at discriminating between USVs (high percentage of green squares). Percent discrimination varied across combinations from 9.6% to 100%. The Mann–Whitney *U* test found no significant differences between socially housed and isolated mice for any USV-USV discrimination combination.

### Multidimensional scaling analyses

**Figure 7.**
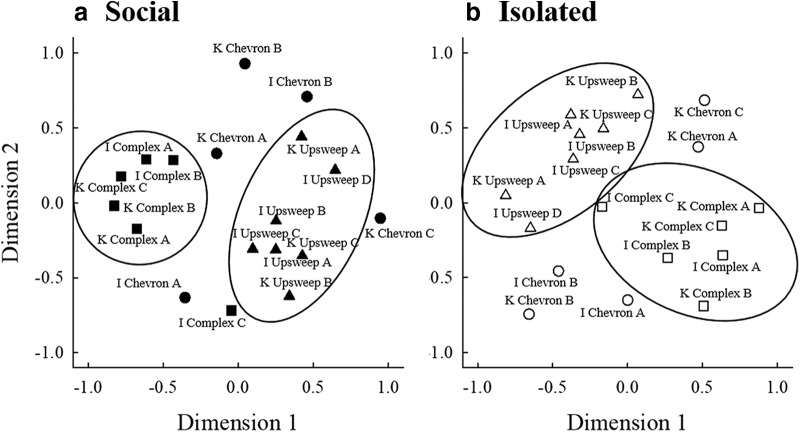
Multidimensional scaling analysis for (***a***) social (filled symbols) and (***b***) isolated mice (open symbols) for chevron (circles), complex (squares), and upsweep (triangles) USVs. Large circles represent researcher-created categories of USVs in similar perceptual spaces.

**Figure 8.**
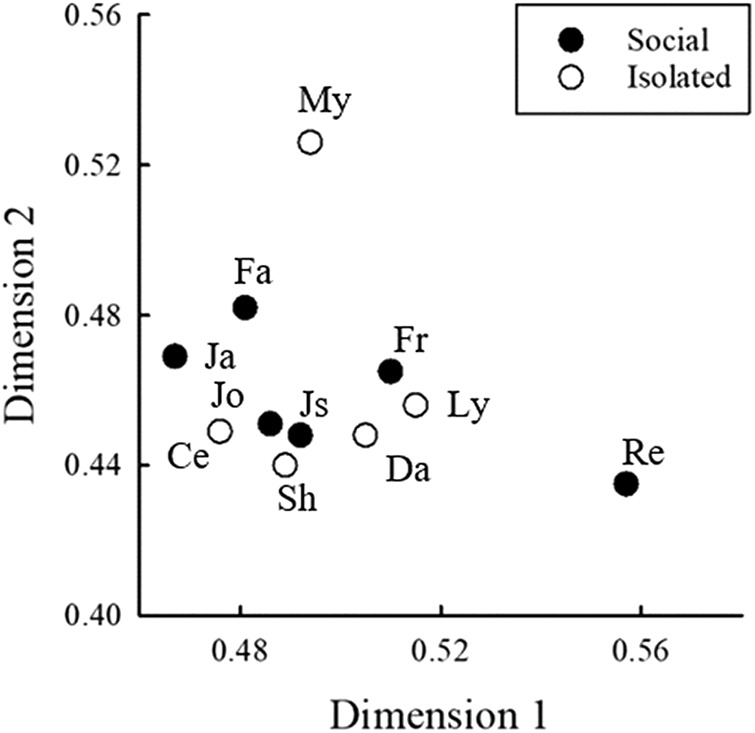
Map of the individual subject weights of social (filled symbols) and isolated (open symbols) mice that contributed to the multidimensional scaling analysis. Subjects close together used similar attributes when discriminating between USVs.

Individual matrices were created from each mouse’s percent correct discrimination of every USV target from every USV background. These matrices were used to calculate multidimensional maps of USV perception in the two groups of mice (Fig. 7). Due to the asymmetry of the matrices (percent corrects were not equal in corresponding cells above and below the diagonals), the maps were created using a PROXSCAL analysis using the full matrix from each mouse. The two-dimensional maps accounted for 91% of dispersion in social mice and 90% of dispersion in isolated mice. In general, for mice in both housing conditions, perceptual maps generally grouped USVs within categories close together, especially the complex and upsweep USVs. There are differences between the two maps, suggesting that overall perception of the USVs differs between the two groups of mice, although that difference cannot be quantified using this technique. Individual weight maps depict the similarity of responses based on mouse identity (Fig. 8). There were no clear separations of mice from the two housing conditions. That is, individual subjects from both groups used similar features of USVs for discrimination.

### Spectrotemporal similarity

**Figure 9.**
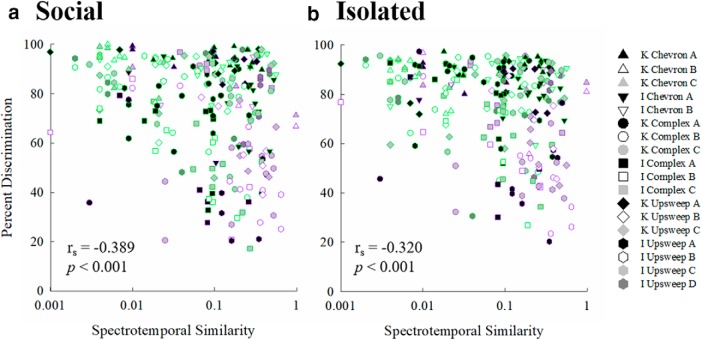
Percent correct discrimination as a function of spectrotemporal similarity by (***a***) social and (***b***) isolated mice. Symbols represent mean discrimination for each target in all 18 conditions. Each symbol represents a different background USV. Symbol edges are purple for within category discriminations and green for across category discriminations. Spearman’s rank order correlation coefficients are shown for each group of mice.

Spectrotemporal similarity was computed using Raven Pro software (v.1.5, Cornell Lab of Ornithology) using the Batch Correlator function, which computed the similarity of the 18 USVs against each other (see *Raven Pro 1.4 User’s Manual*, pp 221–224 for calculation). Similarity ranged on a scale from 0 to 1, with 0 being not at all similar and 1 being identical. The role of spectrotemporal similarity on discrimination performance was examined using Spearman’s rank-order correlation (Fig. 9). There was a significant negative correlation between spectrotemporal similarity and percent discrimination in both socially isolated (*r*_s_^2^ = –0.320, *p* < 0.001) and socially experienced mice (*r*_s_^2^ = –0.389, *p* < 0.001).

### Categories of USVs

**Figure 10.**
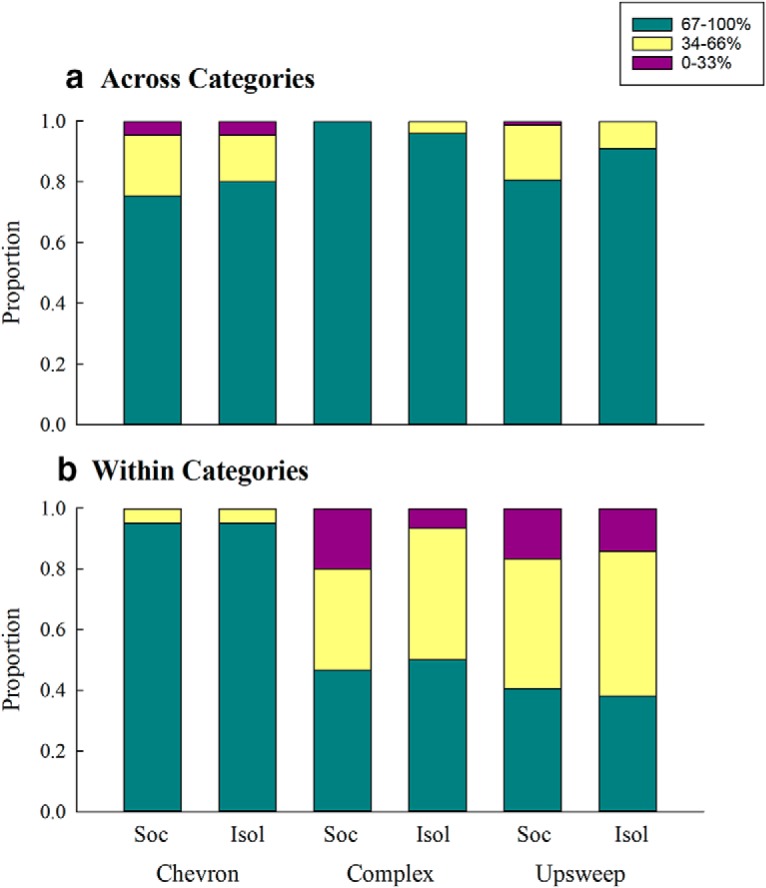
Mean percent discrimination (***a***) across categories and (***b***) within categories, separated by USV (chevron, complex, and upsweep) and housing condition (socially housed (Soc) and isolated (Isol)). Green portions of the bars represent the best discrimination abilities (67% to 100%), yellow portions represent medium discrimination abilities (34% to 66%), and pink portions represent poor discrimination abilities (0–33%).

For both socially housed and isolated mice, some USVs were difficult to discriminate (e.g., KComplexC vs KComplexA), while other USVs were easy to discriminate (e.g., KChevronA USV targets vs every other USV background). No clear pattern emerged in Figure 6 as to which discriminations were difficult and which were easy, except that discriminations within researcher-defined categories (e.g., complex vs complex) were harder than discriminations across researcher-defined categories (e.g., upsweep vs chevron). When we compared the mean percent discrimination performance for within versus across categories (using the matrices in Fig. 6), this became even more apparent, at least for two of the three categories (complex and upsweep; Fig. 10).

The spectrotemporal correlations in the section above suggest that spectrotemporal similarity plays a role in discriminating between USVs. In Figure 9, the outline color of the symbols represents whether that particular discrimination type was within (purple outlines) or across (green outlines) researcher-defined USV categories. Although discrimination was generally poorer when spectrotemporal similarity was high, Figure 10 shows that this was not the only factor in discriminating between USVs. Most of the lowest percent discrimination values in Figure 9 are outlined in purple, across all spectrotemporal similarities, suggesting categorical perception may also be contributing to performance.

**Figure 11.**
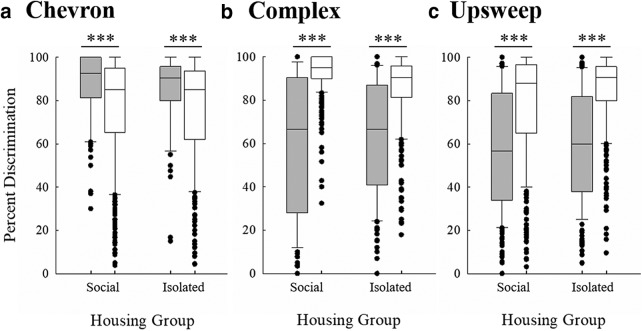
The percentage of correctly discriminated targets from the background USV for within (gray bars) and across (white bars) categories for social and isolated mice tested on (***a***) chevron, (***b***) complex, and (***c***) upsweep USVs. Bars represent upper and lower quartile range of percent discrimination, horizontal lines represent the median, and filled symbols represent data outside the interquartile range; ***p < 0.001.

The categorical perception of USVs was further examined using a Mann–Whitney *U* test for socially housed and isolated mice for each of the three USV categories (Fig. 11). Discrimination of USVs within the same category was significantly worse for socially housed mice for both complex (*U* = 19,551.500, *p* < 0.001) and upsweep (*U* = 27,654.000, *p* < 0.001) backgrounds. Discrimination was also worse within the same category than between categories in isolated mice for complex (*U* = 19,749.000, *p* < 0.001) and upsweep (*U* = 37,322.500, *p* < 0.001) backgrounds. The pattern of discrimination reversed for chevron backgrounds for both social (*U* = 32,317.500, *p* < 0.001) and isolated mice (*U* = 17,024.000, *p* < 0.001), with mice discriminating targets within the chevron category better than they discriminate chevrons versus USVs from other categories. These results are similar to the multidimensional scaling analyses results, where the mice placed the complex and upsweep USVs into separate perceptual spaces, while the chevrons were located throughout the maps. Thus, it appears that the chevron USVs were not perceived as a single category, while the mice may have perceived the complex and upsweep USVs categorically.

## Discussion

The goal of this experiment was to determine whether the perception of USVs was affected by social isolation in mice. It is unclear whether social isolation caused deficits in learning the operant task using simple auditory stimuli (16-kHz pure tone), but isolated mice required significantly more days to learn the task than socially housed mice. There were early deficits in USV discrimination for mice in both housing groups, which disappeared after a few sessions. This deficit was more pronounced in isolated mice, as they required significantly more days to complete the first USV discrimination condition than socially housed mice. It is worth investigating further whether social isolation alters acoustic processing in general, or whether it could be limited to natural signals, such as USVs. If social isolation produces significant stress or alters motivation levels of the mice, there should be no difference between natural and synthetic signal perception, just differences in task learning. However, the effects of this type of “social buffering” (Sullivan and Perry, 2015) on auditory perception are largely unknown.

The multidimensional scaling analysis (Fig. 7) showed that mice grouped USVs into categories that generally agree with the categories we created for the stimuli in this experiment. It is important to note that the location of each USV relative to all the others is the main point of consideration, and the exact location of the USVs is not relevant for the interpretation of the maps. The greatest differences in the perception of USVs between housing conditions emerged within the chevron category. For both socially housed and isolated mice, chevron USVs were not localized as a single group, but were located between and around the complex and upsweep categories; however, the location of these USVs within the perceptual space was not the same for isolated and socially housed mice. This suggests that isolated and socially housed mice perceived USVs classified as chevrons a priori much differently, despite these USVs having similar spectrotemporal “shapes.” In contrast, mice in the two housing conditions showed similar patterns of mapping for the complex and upsweep USVs. The lack of a distinct chevron group in the maps suggests that the criteria used by researchers to define categories are not always accurate; mice may attend to characteristics of the USVs that researchers currently do not take into account when creating categories.

Mice from both housing conditions used similar parameters of USVs to discriminate between stimuli. The dimensions of both perceptual maps were compared to the parameters of the stimuli and general patterns emerged (exceptions to these patterns may exist, and we have no way of knowing from these results whether these are the parameters that the mice are actually using for discrimination). In socially housed mice, dimension 1 of the perceptual map could correspond to the starting frequency of the USVs. The negative side of this dimension corresponds to USVs with higher starting frequencies, and the positive end contains USVs with lower starting frequencies. Alternately, dimension 1 could correspond to frequency modulation. The negative side contains “complex” USVs with a larger number of changes in frequency direction within the USV and the positive section contains “simple” USVs with fewer changes in frequency direction across the USV. Dimension 2 may be related to the duration of the stimuli, as USVs with longer durations are generally aligned with the positive portion of this dimension and USVs with shorter durations generally aligned with the negative portion of this dimension. Dimension 1 for isolated mice likely corresponds to the frequency modulation of the USVs. The negative portion of dimension 1 contains simple USVs with fewer changes in frequency direction, whereas the positive portion contains complex USVs with more changes in frequency direction. Dimension 2 in the isolated mice’s perceptual map appears to relate to duration, with USVs of shorter durations aligned with the positive portion and longer durations aligned with the negative portion of this dimension. There may be other parameters used by the mice to aid in their discrimination that could align with these dimensions as well.

The results from the two socially housed mice who were new to psychophysical testing (Fr, Js) did not differ from three of the four mice who had previously participated in a USV discrimination task (Fa, Ja, Jo), as shown in the individual weight map (Fig. 8). Additionally, the individual weight map shows that socially housed and isolated mice generally perceived USVs the same way. The majority of mice in both groups were clustered together, providing evidence for similar perceptual patterns for USVs.

This experiment is only the third investigation of USV discrimination using natural stimuli from adult mice and behavioral methods. Using similar methods, Holfoth et al. (2014) found that, in mice, the beginning of the USV was more important for discrimination than the middle or end of the USV, paralleling human word recognition. In the present experiment, the very slight differences in the stimulus presentation rhythm when a background USV was alternated with the target USVs are unlikely to be noticed, as the mice in the Holfoth et al. (2014) experiment showed extremely poor performance (∼20% correct) when discriminating a whole USV from the first third of a cropped USV. This was found to be unique to USVs, as discriminating between a USV and a short pure tone was easier (but mice still performed at only 60% correct). Neilans et al. (2014) found that mice could discriminate between USVs of five categories. The results from Neilans et al. (2014) suggested mice relied on spectrotemporal similarity of USVs to aid in their discrimination. When two USVs were highly spectrotemporally correlated, they were more difficult to discriminate. The current experiment aimed to expand on the results of Neilans et al. (2014) by using multiple USVs per category, as well as to determine whether social experience changes how mice rely on spectrotemporal similarity to discriminate between USVs. Mice in both housing conditions showed a negative correlation between spectrotemporal similarity and USV discrimination, agreeing with the results of Neilans et al. (2014). In socially isolated mice, spectrotemporal similarity accounts for 32.0% of the variance observed in discrimination performance. Similarly, spectrotemporal similarity accounts for 38.9% of the variance in socially housed mice’s discrimination ability, slightly more than in isolated mice. This suggests that mice rely on spectrotemporal similarity, at least in part, to help discriminate between USVs.

Finally, this experiment was the first to test whether mice perceive their USVs in accordance with the researcher-defined categories that are found throughout the literature. Categorical perception is characterized by poor discrimination of within-category stimuli and better discrimination of across-category stimuli. We investigated whether mice showed this pattern for discrimination of researcher-defined USV categories. Mice in both housing conditions showed increased discrimination performance when the target and background USVs were in different categories compared to when both target and background USVs were within the same category (Figs. 9–11). Although it has previously been demonstrated that maternal female mice show categorical perception of pup calls (Ehret and Haack, 1981; Ehret, 1992), this is the first step in demonstrating that mice may show categorical perception of adult USVs, providing further evidence that mice are able to use their USVs to communicate important information. Although much more investigation is required to determine how USVs are used in adult communication, it is clear that mice are able to discriminate between USVs, even those within the same researcher-defined categories (e.g., complex vs complex). The results of this experiment provide evidence that mice could be using USVs for communication, since the first criterion is discriminating between different USVs. Further, both the categorical perception analyses and the multidimensional scaling analyses suggest that some researcher-defined categories of USVs are better than others. Discriminating among chevron USVs did not follow the same trend as discrimination of the other two categories.

The findings of the present experiment demonstrate how perception of communication signals is and is not affected by social experience. Socially housed and isolated mice relied, at least in part, on different parameters of USVs to aid in discrimination. This difference is illustrated by dissimilarities between the perceptual maps of mice from the two housing conditions. The results suggest that socially housed mice are probably more attentive to specific features of USVs (e.g., start frequency) compared to isolated mice, who attended to more global features of the targets (e.g., duration, overall frequency modulation). The categorical perception analyses suggest that any differences in the perception of USVs between housing groups are minor, and do not affect the general classification of various acoustic signals. The differences in results between the complex and upsweep USVs (seemingly accurately defined mouse perceptual categories) and the chevron USVs (seemingly inaccurately defined mouse perceptual categories) suggest that mice may be attending to characteristics present in USVs that humans are unable to recognize.

## Synthesis

Reviewing Editor: Tatyana Sharpee, The Salk Institute for Biological Studies Decisions are customarily a result of the Reviewing Editor and the peer reviewers coming together and discussing their recommendations until a consensus is reached. When revisions are invited, a fact-based synthesis statement explaining their decision and outlining what is needed to prepare a revision will be listed below. The following reviewer(s) agreed to reveal their identity: Robert Liu, Peter Heil. Note: If this manuscript was transferred from JNeurosci and a decision was made to accept the manuscript without peer review, a brief statement to this effect will instead be what is listed below.

This study reports longer training times for animals that were socially (but not acoustically) isolated and also differences in discrimination times between and across different types of ultrasound vocalizations. The results build on previous work and can provide a nice addition to the field. There were a number of both major and minor concerns raised by the reviewers. Addressing these concerns is critical to improve the impact and clarity of the manuscript.

Reviewer 1:

Screven et al report results of training mice to discriminate ultrasonic vocalizations (USVs) using a nose-poke paradigm to detect a change in a stream of USVs from a background USV. Training results are reported for 18 different USVs from 3 different types of spectrotemporally distinct categories of USVs (upsweep, chrevron and complex). The most interesting new result of this study concern the shorter time to initially train animals that were socially housed before training began, vs. those that were isolation housed - a result that held only for USVs and not pure tones. This is perhaps suggestive of an initially coarse perception of USVs by isolated animals, which can be overcome through experience or operant training. A second interesting finding is that irrespective of the housing condition, a subject's discrimination between USVs within the same category is more difficult than between USVs across categories, suggestive of a perception of USVs that may be categorical. The results are a nice extension of the lab's previous work to assess USV discrimination by mice (Neilans et al, 2014). They are not particularly surprising, but they do add concrete evidence for the community about whether experimenter-delineated spectrotemporal categories (e.g. upsweep, etc.) are intrinsically perceptually distinct from some other spectrotemporal categories for mice.

I would suggest, however, that the authors address a few issues to improve the clarity and usefulness of this manuscript for the community, as well as several minor points.

Main comments

1) It would not be that surprising to find that operant training per se can lead to the same degree of discrimination in the social and isolated groups. The few cases where a significant difference was found for particular contrasts could simply be statistically significant by random chance, given the high number of comparisons. In fact, it does not appear that multiple comparisons are taken into account in the statistical analyses for discrimination performance, and some of the contrasts in Figs 4-6 marked as significant look only marginally different from other cases that are not. If they are not significantly different once multiple comparisons are taken into account, then it may not be worth commenting on. In fact, as it stands, there is no explanation as to why those particular contrasts would be different between socially housed and isolated mice.

2) It would be helpful to break down the discrimination by category result (Fig 10) according to the different spectrotemporal categories. Presumably there would be an alignment with the multidimensional scaling results, but it is hard to tell from the current figure and text.

3) The work misses some relevant previous work for points they make. For example, even though this work may be the first to show within vs. between category discrimination differences for natural USVs, it is not the first to show categorical perception of ultrasounds in mice - a point that appears to be ignored. Ehret and colleagues have demonstrated categorical perception of ultrasonic noise and tones in their frequency, bandwidth and duration. Also, the point about plasticity from social housing (starting Line 391) is reasonable, but then to talk only about potential plasticity in subcortical tonotopic maps misses the large literature (partially cited elsewhere in the paper) demonstrating plasticity for USVs in auditory cortex.

Minor points

1) It is useful to see the spectrograms of all 18 USVs used as backgrounds and targets for the specialist to be able to better understand the details of the discrimination results. These could be plotted smaller and grouped by spectrotemporal category.

2) Pure tones are first used to train animals to perform the task. No information is given about what tones were used for this. Were they ultrasonic as well?

3) It seems like “condition number” in Fig 3 refers to the serial order in which animals were challenged with background/target combinations, irrespective of what the specific combination was for a specific animal. If that is the case, it would be helpful to state that clearly. Also, to have more transparency to interpret the various specific results, it would be helpful to know (maybe in a table) the specific sequence of contrasts that each animal faced in each of the 18 conditions.

4) Line 247 -is the “p > 0.005” a typo, or was some multiple comparison criterion used?

5) Line 181-182. Was detection of >80% of targets with <20% false alarm in one 200-trial session taken to be the “criterion” for being trained for that background+target “condition”? That is not explicitly defined, even though the term “criterion” and “condition” are used throughout, and it would be helpful to state that definitively.

6) Line 345-349. The statement about Dimension 2 corresponding to the end frequency, yet having an inverted-U shape seems contradictory. The reference to the distortion product explanation needs some unpacking.

7) Lines 368 and 380. “If” should be “whether.”

8) Line 389 - there is already plenty of evidence that mice use USVs to communicate, for example between infants and adult caretakers. As currently phrased, this sentence (and in fact the whole paragraph) seems to discount that work. There are advances here in the context of adult USV perception and communication, but that should be explicitly stated as a qualifier for the advances made in this work.

Reviewer 2:

In this behavioral study, Screven and Dent examine whether the discriminability of ultrasonic vocalizations (USVs) of mice by mice is affected by social isolation. They studied female mice that were socially (but not acoustically) isolated from their litter right after weaning by raising them in individual cages (n=5) and mice that were housed socially (n=6) by raising them in groups of four within a cage. All individually housed mice were unexperienced with the behavioral paradigm (an operant go/no-go procedure) while four of the six socially housed mice were experienced, creating a potential confound of task-experience with housing. After some initial training on a pure tone discrimination task (where individually and socially housed mice performed similarly), the eleven mice were tested with respect to their ability of discriminating 18 USVs from one another.

The authors show results that indicate that the individually housed mice showed early deficits in performance which are interpreted as deficits in perception (Figure 3). However, it is not explained how these data were obtained (see comment to lines 218f and 241ff below) so the reader cannot judge the validity of the claim. The overall (or later?) discrimination performance was similar for the two groups of mice (Figures 4-8). Discrimination performance depended on the spectrotemporal similarity of the USVs (Figure 9), but neither the measure of performance nor that of spectrotemporal similarity is adequately explained in manuscript. Finally, for both groups of mice, discrimination performance is better for USVs from different experimenter-defined vocalization categories than for USVs from within such a category (Figure 10).

Once a number of issues (including issues concerning the clarity of writing) have been fixed, so that a reader can actually understand what has been done, it remains to be seen whether the conclusions are justified. The data might be of interest to some.

Major issues that need better explanation

Line 190: When the target USVs alternated with the background USVs, was the silent interval between consecutive USVs also kept at 200 ms? If so, the animals may have used a change in rhythm as an (additional) cue for discrimination in cases where the durations of background and target USVs differed. Can this be ruled out?

Lines 201ff: It is unclear how many trials a mouse performed within one session. How many sessions did a mouse perform per day? Why were the 17 target USVs divided into three groups (two of seven and one of three)? Am I correct in assuming that, in this way, there were approximately 200/7 trials for each of the first 14 USVs and 200/3 trials (i.e., more than twice as many) for each of the last three USVs?

Lines 218f and 241ff: The requirement for a condition to be considered “complete” by the authors is not specified anywhere. Without this information, this reviewer has no clue what is plotted in Figure 3. Obviously, a complete session does not correspond to a particular number of trials. Am I right? If “Days per Condition” is some measure of the total time required to complete a given number of trials, then the longer time taken by the isolated mice for the initial conditions compared to the social mice might be a consequence of differences in the speed with which animals executed the task (e.g., due to differences in motivation) rather than reflecting true differences in perception or discrimination ability.

Lines 219 and 242ff: In any event, “the number of days required to complete each condition” cannot be normally distributed (because those numbers cannot be negative) and therefore a key prerequisite for the use of an ANOVA is violated.

Line 246: It is unclear how discrimination performance and percent discrimination were actually computed. The legend to Figure 10 implies that percent discrimination simply represents the percentage of targets correctly discriminated from the background USVs (i.e., the percentage of hits). If true, the percent discrimination will be affected by the criterion of the mouse and will be reflected in the false-alarm rate. Why not apply signal detection theory and use a measure of sensitivity that attempts to correct for criterion, such as d-prime?

Lines 260-279: The lengthy and detailed description of the statistical results could be omitted. Isn't it noteworthy that the difference in performance between social and isolated animals is significant when, for example, K Chevron B is the background and K Chevron A is the target, whereas the difference is not significant when K Chevron A is the background and K Chevron B is the target? The same asymmetry holds for all other comparisons marked with an asterisk in Figure 4-6. I would therefore be inclined to interpret the significant differences as spurious results (type-I errors). This is also supported by the fact that you find significant (p<0.05) differences in performance in only 12 of the 306 comparisons. This corresponds to about 4%, close to the 5% expected by chance with a p<0.05 criterion if performance of isolated and social mice did not differ. I agree with the authors' summary on lines 282f.

I am wondering whether the results in Figures 4-6 could be plotted in a more compact way that would also highlight the asymmetries mentioned above. One possibility would be to show them as a two-dimensional matrix with USVs 1-18 shown in rows and columns. Squares below and above the diagonal could be used to represent performance when a given USV is the background and when it is the target, respectively. The (median) performance itself could be color coded and asterisks used to mark significant differences.

Lines 297ff: Spectrotemporal similarity. There is no mention in the manuscript of how spectrotemporal similarity was computed. This also needs to be done. Furthermore, the linear regression is certainly not a good model to describe the relationship between spectrotemporal similarity and percent discrimination (also not explained how it was computed; see above). I suggest computing, for example, Spearman's rank correlation coefficients instead to assess whether there are monotonic relationships. I also suggest plotting spectrotemporal similarity using a logarithmic axis to obtain better resolution for low similarity.

I am not convinced by the conclusion of the authors (e.g., lines 384ff) that mice do show categorical perception of their USVs. Presumably, USVs within an experimenter-defined category are more similar to one another than USVs from different experimenter-defined categories. Can't the data in Figure 10 simply be explained by the effects of spectrotemporal differences on discrimination performance shown in Figure 9?

Minor issues

I am under the impression that the term “condition” is used inconsistently by the authors. The term “condition number” in Figure 3 might be misleading, because the actual conditions (e.g., USV 1 vs. USV 2) underlying each condition number can be different for different mice.

Lines 58 and 61: Changes with what?

Line 71: “vary”. With what? Do you mean “differ”?

Lines 84ff: I don't understand what you want to say here.

Line 86: “changes”. Do you mean “differences”?

Line 103: “This difference...”. Which difference?

Line 105: Insert “pup-unexperienced” before “virgin”.

Line 106: Insert “differences in” before “spiking activity”.

Line 124 and elsewhere: I'd prefer if the mice were identified by numbers or letters rather than names.

Line 128: “F Exposed mice”. I don't understand.

Line 148: “Three mice lived with one vocalizer...”. Please specify the mice and the vocalizer.

Line 149: What are “testing mice”?

Lines 164ff: I think it would be useful if you would introduce the USVs, their “renditions”, and their nomenclature here.

Lines 165ff: The Chevron examples shown in Figure 2 are much longer than the mean Chevron duration reported.

Line 167: “two changes in frequency”? Do you mean “two changes in the direction of frequency modulation”?

Line 169: “constant”? Do you mean “monotonic”? The example “upsweep USV” shown in Figure 2e, however, shows a downward frequency modulation followed by an upward frequency modulation.

Line 171: The intensity or level probably varies over time, no? So what does 50 dB SPL then specify?

Line 179: Please specify the pure tones (frequency, duration, rise/fall; level of high-level target; repetition rate, etc.).

Lines 180f: “USVs” should read “pure tones” here, right?

Line 187: Delete “stimulus” before “USVs”.

Line 194: Is “Ensure” a commercial product? If so, please identify. I presume the reinforcement was delivered via the dipper. Correct?

Lines 196 and 206: Please specify “immediately”.

Line 199f: Replace “Each block was randomly generated so...” by “The sequence of ‘go’ and ‘no-go’ trials within each block was random except for the constraint that...”. I would try to avoid the term “sham trial”.

Line 207: I don't understand how you arrive at the conclusion that chance performance is represented by the false alarm rate?

Line 208f. “This criterion ensured...”. Delete this wrong statement.

Line 237: “Length”? Do you mean “Number ”?

Line 239: “with respect to” rather than “on”.

Line 240f: Suggest showing the two distributions, rather than just providing the medians, of the number of days required to complete training, so one can appreciate whether the two distributions have similar shape (a prerequisite for the Mann-Whitney U-test). Identify the individuals.

Lines 246ff: Do the results of the posthoc pairwise comparisons performed here include corrections for multiple testing and, if so, which ones? Which comparison test was used? Holm-Sidak?

Lines 284ff: Multidimensional scaling analysis. I must admit that I have no (good) understanding of the PROXSCAL analysis. So, I don't know for example whether there is any significance to the result that, in the two-dimensional plots shown in Figure 7, K Chevron B is located at approximately 0, 1 in socially housed animals and far away, at -0.6, -0.8, in individually housed mice. It would perhaps be interesting, for other ignorant readers, to see these maps for the data split, say in half, for each group to see how reproducible or reliable the patterns are.

Line 294ff: Similarly, I have no good understanding of the meaning of Figure 8, but I agree with the authors that there appears to be no clear separation of the groups.

Line 295: The reference should probably be to Figure 8 rather than Figure 9.

Line 310: What is a “training rate”??

Line 311: “discrimination” rather than “perception”?

Line 314: Onset of what?

Line 348f: I don't understand how the authors arrive at this conclusion here.

Lines 375f: “...with spectrotemporal similarity playing a slightly larger role in socially housed mice than isolated mice.” I think this is an over-interpretation of the data, given the large scatter of the data points around the linear regression lines.

Lines 391-402: This is all highly speculative.

Line 451: Please correct page numbers for this reference.

Lines 464f: There are no asterisks in Figure 3.

Lines 470, 476, and 482: Are you sure the error bars in Figures 4-6 are confidence intervals rather than marking the interquartile ranges? In addition, it is impossible in most cases to unequivocally assign error bars to symbols, because they overlap. Please consider introducing some horizontal offset between open and filled symbols, if you want to retain these figures.

Line 497: I would avoid the term “outlier”. Why not say, e.g., “data outside the interquartile range”?

Figure 1 is not really needed.

Figure 2 should use a reversed gray scale or a color scale.

Figures 4-6 could be condensed into one panel, as suggested above.

Figure 9 should use logarithmically scaled x-axes. Please remove regression lines and show Spearman rank order correlation coefficients instead.
